# A Novel Nomogram for Predicting Postoperative Liver Failure After Major Hepatectomy for Hepatocellular Carcinoma

**DOI:** 10.3389/fonc.2022.817895

**Published:** 2022-03-14

**Authors:** Zhengqing Lei, Nuo Cheng, Anfeng Si, Pinghua Yang, Guangmeng Guo, Weihu Ma, Qiushi Yu, Xuan Wang, Zhangjun Cheng

**Affiliations:** ^1^Hepato-Pancreato-Biliary Center, Zhongda Hospital, School of Medicine, Southeast University, Nanjing, China; ^2^Department of Hepatic Surgery IV, the Eastern Hepatobiliary Surgery Hospital, Naval Medical University, Shanghai, China; ^3^School of Medicine, Nanjing University of Chinese Medicine, Nanjing, China; ^4^Department of Surgical Oncology, Qin Huai Medical District of Jinling Hospital, Nanjing Medical University, Nanjing, China; ^5^Department of Minimally Invasive Surgery, the Eastern Hepatobiliary Surgery Hospital, Naval Medical University, Shanghai, China

**Keywords:** post-hepatectomy liver failure, hepatocellular carcinoma, major hepatectomy, nomogram, prediction model

## Abstract

**Background:**

Post-hepatectomy liver failure (PHLF) is the most common cause of mortality after major hepatectomy in hepatocellular carcinoma (HCC) patients. We aim to develop a nomogram to preoperatively predict grade B/C PHLF defined by the International Study Group on Liver Surgery Grading (ISGLS) in HCC patients undergoing major hepatectomy.

**Study Design:**

The consecutive HCC patients who underwent major hepatectomy at the Eastern Hepatobiliary Surgery Hospital between 2008 and 2013 served as a training cohort to develop a preoperative nomogram, and patients from 2 other hospitals comprised an external validation cohort. Least absolute shrinkage and selection operator (LASSO) logistic regression was applied to identify preoperative predictors of grade B/C PHLF. Multivariable logistic regression was utilized to establish a nomogram model. Internal and external validations were used to verify the performance of the nomogram. The accuracy of the nomogram was also compared with the conventional scoring models, including MELD and ALBI score.

**Results:**

A total of 880 patients who underwent major hepatectomy (668 in the training cohort and 192 in the validation cohort) were enrolled in this study. The independent risk factors of grade B/C PHLF were age, gender, prothrombin time, total bilirubin, and CSPH, which were incorporated into the nomogram. Good prediction discrimination was achieved in the training (AUROC: 0.73) and validation (AUROC: 0.72) cohorts. The calibration curve also showed good agreement in both training and validation cohorts. The nomogram has a better performance than MELD and ALBI score models.

**Conclusion:**

The proposed nomogram showed more accurate ability to individually predict grade B/C PHLF after major hepatectomy in HCC patients than MELD and ALBI scores.

## Introduction

Hepatocellular carcinoma (HCC) is the most common liver cancer, accounting for approximately 80% of all primary liver malignancies. Partial hepatectomy (PH) remains mainly curative-intent treatment for HCC. Although postoperative morbidity and mortality have decreased over the past decades with advances in surgical techniques and perioperative management (1, 2), they remain high in HCC patients who underwent major hepatectomy. Previous studies have revealed that post-hepatectomy liver failure (PHLF) is one of the most seriously complications and the main cause of mortality particularly in patients undergoing major hepatectomy (3–7). Therefore, accurate preoperative prediction of the risk of PHLF is essentially important for patient selection and perioperative management.

Conventionally, some clinical scoring models, including Child–Pugh score, Model for End-Stage Liver Disease (MELD) (8), and albumin–bilirubin (ALBI) grade (9), were used to evaluate preoperative liver function reserve. Recently, these liver function reserve models were adopted to predict PHLF and exhibited some predictive ability (10–13). Unfortunately, the accuracy of these models in predicting PHLF is limited. In our previous study, we validated and compared the predictive ability of 6 liver function reserve models for PHLF in patients with HCC after major hepatectomy. Our results revealed that although the ALBI score shows more accurate predictive ability, the areas under the receiver operating characteristic curves (AUROC) was only 0.64 (14), which indicated a limited accuracy. Therefore, development of a more accurate predictive model for PHLF is needed.

Recently, a few predictive models have been established for PHLF in HCC patients and showed certain predictive ability (15–19). However, most of these models incorporated both pre- and postoperative predictors which might reduce the validity of preoperative prediction. Moreover, few models were developed based on data from major hepatectomy. Considering the relatively high incidence of PHLF and, more importantly, of preoperative assessment for major hepatectomy, development of a predicting model for PHLF prior to surgery is vital to avoiding PHLF, especially for HCC patients considered undergoing major hepatectomy.

In this study, we aimed to determine the preoperative risk factors of PHLF and construct a nomogram based on the identified predictors to individually predict PHLF after major hepatectomy in HCC patients and compare with conventional models to evaluate the accuracy of the nomogram in predicting PHLF.

## Patients and Method

### Study Design

**Figure 1:**
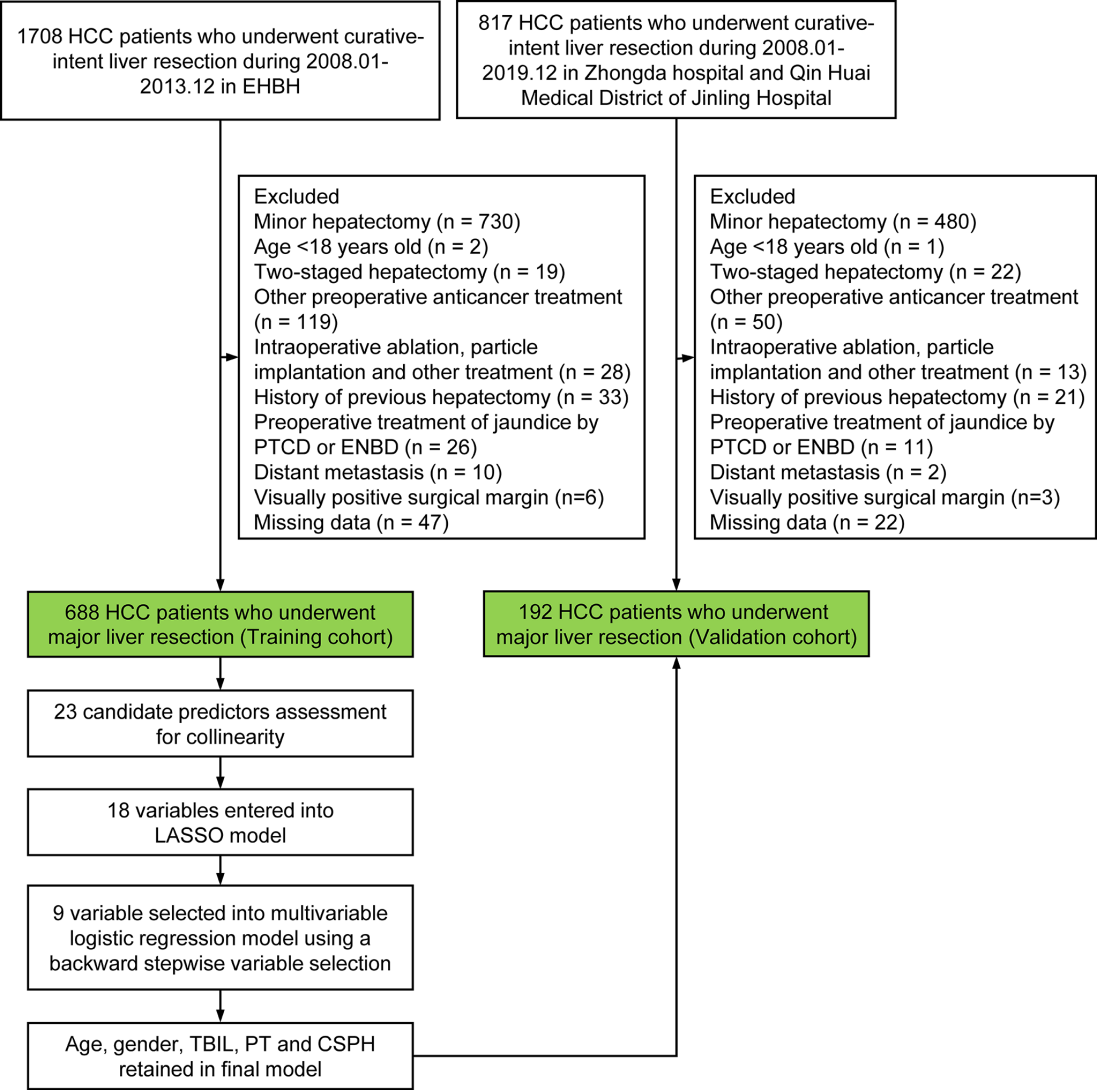
Flowchart of the study. PVE, Preoperative portal vein embolization; TACE, transcatheter arterial chemoembolization; ALPPS, associating liver partition and portal vein ligation for staged hepatectomy;PTCD, percutaneous transhepatic cholangial drainage; ENBD, endoscopic nasobiliary drainage; HCC, hepatocellular carcinoma; LASSO, least absolute shrinkage and selection operator; TBIL, total bilirubin; PT, prothrombin time; CSPH, clinically significant portal hypertension.

Multicentric data of HCC patients who underwent major hepatectomy at the Eastern Hepatobiliary Surgery Hospital in Shanghai (EHBH) from January 2008 to December 2013, Zhongda Hospital, Southeast University in Nanjing (ZDH), and Qin Huai Medical District of Jinling Hospital in Nanjing (QHMD) between January 2008 and December 2019 were retrospectively collected and analyzed. The data sets from EHBH were used to develop a nomogram (training cohort), and the data sets from ZDH and QHMD were used for external validation of the nomogram (validation cohort). The flowchart of the study design is shown in **Figure 1**.

The inclusion criteria were as follows: 1) HCC pathologically confirmed and 2) underwent laparoscopic or open major hepatectomy. The exclusion criteria were as follows: 1) age <18 years old; 2) received two-staged hepatectomy after preoperative portal vein embolization (PVE) or associating liver partition and portal vein ligation (ALPPS); 3) received preoperative transcatheter arterial chemoembolization (TACE); 4) conducted intraoperative ablation, particle implantation, and other treatments; 5) preoperative percutaneous transhepatic cholangial drainage (PTCD) or endoscopic nasobiliary drainage (ENBD); 6) history of previous hepatectomy; 7) distant metastasis; 8) visually positive surgical margin; and 9) missing data.

The study was approved by the Institutional Ethics Committee of each of the involved hospitals. Informed consent was routinely obtained before surgery. The study was censored on June 30, 2021.

### Preoperative Workup

All patients underwent contrast computed tomography (CT) or magnetic resonance imaging (MRI) to evaluate liver tumors, and the presence of arterial enhancement and portovenous washout were considered typical radiological features of HCC. Selection for hepatectomy was based on assessment of the patient’s general condition, tumor burden, liver function, and future liver remnant (FLR) volume. In patients with cirrhosis, a minimum cutoff FLR volume of 40% was used. Assessment of liver function was based on Child–Pugh score, MELD score, and ALBI grade. Upon referral, laboratory tests including complete blood cell count, coagulation profile, liver and renal function, plasma levels of alpha-fetoprotein (AFP), hepatitis B and C virus serology, and HBV deoxyribonucleic acid (HBV-DNA), a chest X-ray, or CT and gastroscopy were routinely performed.

### Definitions of Variables

The primary end point of this study was grade B or C PHLF according to the International Study Group on Liver Surgery Grading (ISGLS) (7). Major hepatectomy was defined as resection of three or more Couinaud’s liver segments (20). Postoperative complications were consistent with the Clavien–Dindo classification (21). Clinically significant portal hypertension (CSPH) was defined as the presence of esophageal varices detected by endoscopy or significant splenomegaly (major diameter >12 cm) with a platelet count <100,000/mm^3^ (22). The Charlson Comorbidity Index was calculated based on the weights listed by Charlson et al. (23).

### Statistical Analysis

Variables considered for the predictive nomogram included patient demographic details, preoperative liver function status, and perioperative clinical and laboratory measurements. The squares of the Spearman correlation coefficients were used to estimate any correlation between candidate variables within the training cohort. The least absolute shrinkage and selection operator (LASSO) method was used to reduce the number of candidate predictors further and select final variables into the multivariable logistic regression model [19]. For this analysis, the penalty term was determined by 10-fold cross-validation and the one yielding the smallest mean square error was chosen as the penalty to use. Multivariable logistic regression was used to estimate variable effects of selected predictors in the training cohort. The nomogram model performance was assessed and compared with the conventional scoring models (MELD and ALBI score), by using AUROC, calibration curves, and DeLong’s test. Statistical analysis was conducted using R software (https://www.r-project.org/) with “rms”, “pROC”, and “calibrationcurves” packages.

## Results

### Baseline Characteristics

A total of 880 HCC patients who underwent major hepatectomy were finally enrolled in the present study. Of them, 688 patients from the EHBH formed the training cohort, and 192 patients from the ZDH and QHMD comprised the external validation cohort. The characteristics of the study cohorts are presented in **Table 1**. No significant differences were observed between the training and external validation cohorts with respect to any patient- or tumor-related covariates. The incidences of grade B/C PHLF were 13.5% and 9.4% in the training cohort and external cohort, respectively.

**Table 1:** Baseline characteristics.

Variable	Total (n = 880)	Training cohort (n = 688)	Validation cohort (n = 192)	p value
**Gender**				1.000
Female	115 (13.1%)	90 (13.1%)	25 (13.0%)	
Male	765 (86.9%)	598 (86.9%)	167 (87.0%)	
**Age** in years	50.4 ± 10.6	50.3 ± 10.6	50.7 ± 10.6	0.590
**Charlson Comorbidity Index**	3.0 (2.0–6.0)	3.0 (2.0–6.0)	3.0 (2.0–4.0)	0.830
**HBsAg**				0.766
Negative	132 (15.0%)	105 (15.3%)	27 (14.1%)	
Positive	748 (85.0%)	583 (84.7%)	165 (85.9%)	
**HBsAg quantification**, Log_10_IU/mL	3.6 (-2.0–3.6)	3.6 (-2.0–3.6)	3.6 (-0.52–3.6)	0.612
**HBeAg**				0.525
Negative	703 (79.9%)	546 (79.4%)	157 (81.8%)	
Positive	177 (20.1%)	142 (20.6%)	35 (18.2%)	
**HBeAg quantification**, Log_10_IU/mL	-0.94 (-2.7–2.92)	-0.94 (-2.7–2.92)	-0.95 (-1.18–2.12)	0.623
**Anti-HCV**				0.745
Negative	867 (98.5%)	677 (98.4%)	190 (99.0%)	
Positive	13 (1.5%)	11 (1.6%)	2 (1.0%)	
**HBV-DNA**, Log_10_IU/mL	3.00 (0.00–8.48)	3.00 (0.00–7.30)	3.00 (0.00–8.48)	0.508
**Preoperative ascites**				1.000
No	831 (94.4%)	650 (94.5%)	181 (94.3%)	
Yes	49 (5.6%)	38 (5.5%)	11 (5.7%)	
**MELD**	7.00 (6.00–20.0)	7.00 (6.00–20.0)	7.00 (6.00–12.0)	0.960
**ALBI** score	-2.75 (-5.30–0.97)	-2.75 (-5.30–0.97)	-2.78 (-3.72–1.46)	0.302
**Child–Pugh class**				0.080
A	851 (96.7%)	661 (96.1%)	190 (99.0%)	
B	29 (3.3%)	27 (3.9%)	2 (1.0%)	
**Child–Pugh score**	5.00 (5.00–9.00)	5.00 (5.00–9.00)	5.00 (5.00–7.00)	0.416
**Cirrhosis**				0.716
No	455 (51.7%)	353 (51.3%)	102 (53.1%)	
Yes	425 (48.3%)	335 (48.7%)	90 (46.9%)	
**CSPH**				0.937
No	757 (86.0%)	591 (85.9%)	166 (86.5%)	
Yes	123 (14.0%)	97 (14.1%)	26 (13.5%)	
**TBIL**, mg/dL	0.81 (0.26–22.8)	0.81 (0.26–22.8)	0.81 (0.32–2.41)	0.729
**ALB**, g/L	41.2 (22.8–68.9)	41.1 (22.8–68.9)	41.6 (27.6–54.6)	0.410
**ALT**, U/L	38.9 (5.00–1131)	39.0 (9.00–1131)	36.5 (5.00–290)	0.197
**AST**, U/L	38.0 (2.00–1059)	38.1 (3.00–1059)	36.0 (2.00–321)	0.227
**PT**, seconds	11.9 (9.50–19.3)	11.9 (9.50–19.3)	12.0 (9.70–15.3)	0.638
**INR**	0.99 (0.80–1.76)	0.99 (0.80–1.76)	1.00 (0.82–1.28)	0.609
**Creatine**, μmol/L	68.0 (3.40–154)	68.0 (3.40–154)	66.0 (33.0–109)	0.196
**PLT**, ×10^9^/L	182 (29.0–663)	180 (29.0–663)	184 (37.0–490)	0.334
**Tumor diameter**, cm	9.0 (5.5–13.0)	9.7 (6.0–13.0)	8.0 (4.6–11.5)	0.001
**Tumor number**				0.004
Single	665 (82.7%)	502 (80.6%)	163 (90.1%)	
Multiple	139 (17.3%)	121 (19.4%)	18 (9.9%)	
**Macrovascular invasion**				0.538
No	650 (73.9%)	512 (74.4%)	138 (71.9%)	
Yes	230 (26.1%)	176 (25.6%)	54 (28.1%)	
**Bile duct tumor thrombus**				0.061
No	856 (97.3%)	665 (96.7%)	191 (99.5%)	
Yes	24 (2.7%)	23 (3.3%)	1 (0.5%)	
**BCLC staging**				0.797
0	7 (0.8%)	5 (0.7%)	2 (1.0%)	
A	540 (61.4%)	425 (61.8%)	115 (59.9%)	
B	103 (11.7%)	82 (11.9%)	21 (10.9%)	
C	230 (26.1%)	176 (25.6%)	54 (28.1%)	
**PHLF**				0.572
Absent	675 (84.0%)	526 (84.4%)	149 (82.3%)	
Present	129 (16.0%)	97 (15.6%)	32 (17.7%)	
**PHLF ISGLS grade**				0.160
0-A	769 (87.4%)	595 (86.5%)	174 (90.6%)	
B-C	111 (12.6%)	93 (13.5%)	18 (9.4%)	

HBsAg, hepatitis B surface antigen; HBeAg, hepatitis B e antigen; anti-HCV, antibody to hepatitis virus C; HBV, hepatitis B virus; DNA, deoxyribonucleic acid; MELD, model for end-stage liver disease; ALBI, albumin–bilirubin; CSPH, clinical significant portal hypertension; TBIL, total bilirubin; ALB, albumin; ALT, alanine aminotransferase; AST, aspartate aminotransferase; PT, prothrombin time; INR, international normalized ratio; PLT, platelet; BCLC, Barcelona-Clinic Liver Cancer; PHLF, post-hepatectomy liver failure; ISGLS, International Study Group of Liver Surgery.

### Morbidity and Mortality

Overall, postoperative 90-day mortality was 4.8% (n = 42/880), 4.7% in the training cohort (n = 32/688) and 5.2% in the validation cohort (n = 10/192); among them, 20 and 5 patients died due to PHLF in the two cohorts, respectively. The PHLF (grade A-C) was observed in 155 (22.5%) and 37 (19.3%) patients in the two cohorts, respectively. The details of postoperative complications are shown in **Table 2**. In brief, 323 and 94 patients developed postoperative complications with a median hospital stay of 15 (4–115) days and 14 (7–35) days, respectively. Major complications occurred in 81 (11.8%) and 23 (12.0%) patients, respectively.

**Table 2:** Surgical procedures and intraoperative characteristics.

Variable	Training cohort (n = 688)	Validation cohort (n = 192)	p value
**Surgical margin**			0.134
R0	677 (98.4%)	192 (100%)	
R1	11 (1.6%)	0	
**Type of hepatectomy**			<0.001
** Open**	688 (100%)	158 (82.3%)	
** Laparoscopic**	0	34 (17.7%)	
**Total clamping time** (min)	20.0 (0.0–400)	20.0 (0.0–337)	0.556
**Blood loss** (ml)	300 (30.0–7000)	400 (50.0–4500)	0.768
**Blood transfusions**	209 (30.4%)	54 (28.1%)	0.607
**Operative time** (hour)	2.0 (0.75–5.2)	2.0 (0.9–5.0)	0.826
**Length of hospital stay** (days)	15.0 (4.0–115)	14.0 (7.0–35.0)	0.266
**Postoperative complication**			1.000
Clavien–Dindo I–II	242 (35.2%)	71 (37.0%)	
Clavien–Dindo III–IV	81 (11.8%)	23 (12.0%)	
**90-day complication**	323 (46.9%)	94 ((49.0%)	0.681
**30-day mortality**	8 (1.2%)	2 (1.0%)	1.000
**90-day mortality**	32 (4.7%)	10 (5.2%)	0.898

### Identification of Candidate Preoperative Predictors for Grade B/C PHLF

Next, we used two different algorithms to identify the most significant preoperative parameters for PHLF. First, we performed variable clustering to exclude the variables that have high collinearity (**Supplementary Figure 1**). As the figure shows, sex and serum creatine are perfectly correlated. Aspartate aminotransferase (AST) and alanine aminotransferase (ALT) levels, hepatitis B surface antigen (HBsAg) and HBsAg quantification, hepatitis B e antigen (HBeAg) and HBeAg quantification, prothrombin time (PT) and international normalized ratio (INR), and Charlson Comorbidity Index and CSPH are also strongly correlated pairs. Thus, AST, HBsAg, HBeAg, INR, serum creatine, and Charlson Comorbidity Index were not included in the LASSO analysis because of their strong correlations with other variables.

Second, we used the LASSO algorithm to further refine a set of 18 factors (**Supplementary Figure 2**). Finally, age, sex, total bilirubin (TBIL), PT, albumin (ALB), PLT, hepatitis C virus antibody (anti-HCV), cirrhosis base on imaging, and CSPH were selected into the multivariable logistic regression.

### Development of Preoperative Nomogram for Grade B/C PHLF

**Figure 2:**
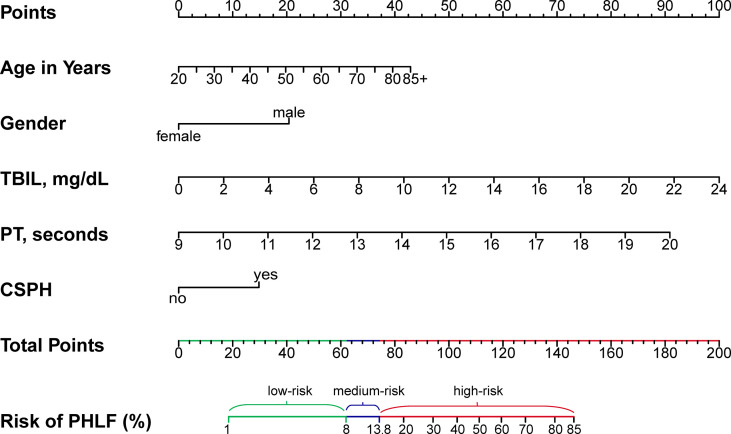
Preoperative nomogram for predicting grade B/C PHLF in HCC patients undergoing major hepatectomy. TBIL, total bilirubin; PT, prothrombin time; CSPH, clinically significant portal hypertension; PHLF, post-hepatectomy liver failure.

A regression model from a set of candidates’ preoperative variables was built by removing predictors based on Akaike information criterion (AIC), in a stepwise manner until there is no variable left to remove any more. Based on the multivariate logistic analysis (**Table 3**), a nomogram integrating age, sex, TBIL, PT, and CSPH was developed. According to the nomogram-predicting probability of grade B/C PHLF, we categorized all the patients to three equal groups as low-risk (predicting probability ≤8.6%), medium-risk (8.6–13.9%), and high-risk groups (>13.9%), corresponding to total nomogram scores of ≤61.9 points, 61.9–74.2 points, and >74.2 points on the full nomogram, respectively (**Figure 2**).

**Table 3:** Univariable and multivariable logistic regression analyses to identify preoperative predictors for PHLF grade B/C in patients with HCC undergoing major hepatectomy.

Variable	Univariable logistic regression		Multivariable logistic regression	
	Odds ratio (95% CI)	p	Odds ratio (95% CI)	p
**Gender, male**	2.85 (1.23–8.38)	**0.012**	2.75 (1.13–8.39)	0.044
**Age in years**	1.03 (1.01–1.05)	**0.010**	1.03 (1.01–1.06)	0.004
**Charlson Comorbidity Index**	1.58 (1.12–2.25)	**0.010**		
**HBsAg, positive**	1.12 (0.61–2.18)	0.732		
**HBsAg quantification, Log10 U/mL**	1.07 (0.92–1.26)	0.379		
**HBeAg, positive**	0.99 (0.56–1.67)	0.973		
**HBeAg quantification, Log10 U/mL**	1.08 (0.85–1.38)	0.539		
**Anti-HCV, positive**	2.52 (0.52–9.10)	0.225		
**HBV-DNA, Log10 U/mL**	1.04 (0.95–1.14)	0.344		
**Preoperative ascites, yes**	1.50 (0.59–3.36)	0.370		
**Cirrhosis, yes**	1.90 (1.22–3.00)	**0.005**		
**TBIL, mg/dL**	1.25 (1.10–1.42)	**0.001**	1.23 (1.10–1.42)	<0.001
**ALB, g/L**	0.94 (0.89–0.99)	**0.013**		
**ALT, U/L**	1.00 (1.00–1.00)	0.616		
**AST, U/L**	1.00 (1.00–1.00)	0.913		
**PT, seconds**	1.58 (1.30–1.92)	**<0.001**	1.51 (1.23–1.86)	<0.001
**INR**	249.81 (23.24–2684.76)	**<0.001**		
**Creatine, μmol/L**	1.01 (0.99–1.02)	0.516		
**PLT, ×10^9^/L**	1.00 (0.99–1.00)	**0.048**		
**Tumor diameter, cm**	1.00 (0.96–1.05)	0.926		
**Tumor number, multiple**	0.89 (0.48–1.58)	0.709		
**CSPH, yes**	2.67 (1.56–4.47)	**<0.001**	2.09 (1.18–3.60)	0.009
**Macrovascular invasion**	1.15 (0.70–1.87)	0.569		
**Bile duct tumor thrombus**	3.66 (1.42–8.77)	**0.009**		
**BCLC**				
** 0**	Ref.	Ref.		
** A**	0.22 (0.03–1.96)	0.156		
** B**	0.19 (0.03–1.78)	0.131		
** C**	0.26 (0.04–2.30)	0.198		

HBsAg, hepatitis B surface antigen; HBeAg, hepatitis B e antigen; anti-HCV, antibody to hepatitis virus C; HBV, hepatitis B virus; DNA, deoxyribonucleic acid; TBIL, total bilirubin; ALB, albumin; ALT, alanine aminotransferase; AST, aspartate aminotransferase; PT, prothrombin time; INR, international normalized ratio; PLT, platelet; CSPH, clinically significant portal hypertension; BCLC, Barcelona-Clinic Liver Cancer; PHLF, post-hepatectomy liver failure. Values with P < 0.05 in the univariable analysis are in bold.

### Internal Validation of the Nomogram

**Figure 3:**
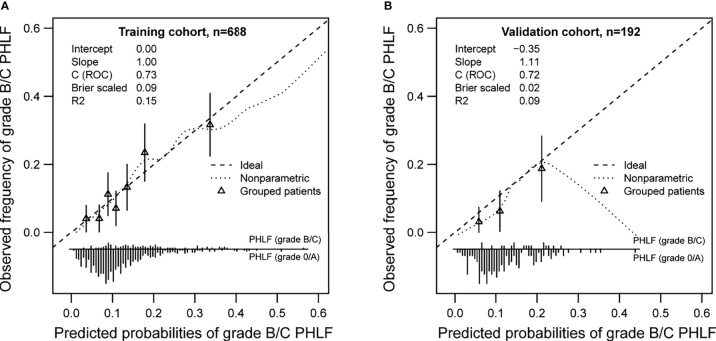
Calibration plots of the preoperative nomogram in training **(A)** and validation cohorts **(B)**.

Bootstrapping with 1,000 repetitions was used for model internal validation. The final multivariable model for grade B/C PHLF showed strong internal validity, with a discrimination C-index of 0.73 (95% CI, 0.69–0.76). Furthermore, the final model demonstrated good internal calibration of observed versus predicted outcomes across a spectrum of risk groups, with a calibration slope of 1.00 and intercept of −0.00, as shown in **Figure 3A**.

### External Validation of the Nomogram

A total of 192 patients comprised the validation cohort. Of these patients, 18 (9.4%) experienced PHLF grade B/C. The apparent AUROC for the nomogram model was 0.72 (0.65-0.78). This model was also well calibrated in the external validation cohort, as would be expected, with a calibration slope of 1.11 and intercept of −0.35 (**Figure 3B**).

### Comparison of Predictive Accuracy for Grade B/C PHLF Among the Nomogram and Conventional Scores

**Figure 4:**
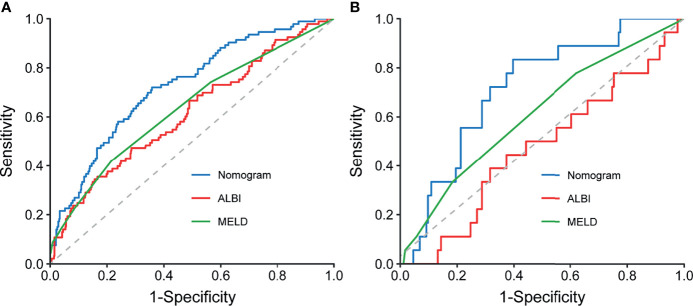
Comparison of ROC curves for the preoperative nomogram, ALBI, and MELD in the training **(A)** and validation **(B)** cohorts. ROC, receiver operating characteristic curves; ALBI, albumin–bilirubin; MELD, Model for End-Stage Liver Disease.

When compared with conventional scores, the nomogram had significant greater discriminatory performance for predicting grade B/C PHLF than MELD and ALBI in the training cohort and external validation cohort (most p < 0.05, p = 0.142 except in the validation cohort when compared with MELD; **Table 4** and **Figure 4**), which was not significantly influenced by inherent heterogeneity in different cohorts.

**Table 4:** Comparison of conventional scores and the nomogram for predicting grade B/C PHLF.

Models	Training cohort			Validation cohort		
	AUROC (95% CI)	*p*	Z statistic	AUROC (95% CI)	*p*	Z statistic
**MELD**	0.64 (0.60–0.67)	0.001	3.281	0.61 (0.54–0.68)	0.142	1.469
**ALBI**	0.62 (0.58–0.65)	0.001	3.199	0.53 (0.46–0.60)	0.032	2.147
**Nomogram**	0.73 (0.69–0.76)	–	–	0.72 (0.65–0.78)	–	–

PHLF, post-hepatectomy liver failure; MELD, model for end-stage liver disease; ALBI, albumin–bilirubin.

p value indicates comparison of nomogram and conventional scores.

## Discussion

In this study, based on data from a large multicentric cohort of HCC patients undergoing major hepatectomy, a nomogram was proposed to individually predict severe PHLF by incorporating preoperative risk factors of PHLF. The AUROC of the nomogram were 0.73 in the training cohort and 0.72 in the external validation cohort, which were higher than those of two conventional liver function reserve models. The accuracy of the nomogram was further confirmed by calibration curve analysis and DeLong’s test. As no tool exists to predict PHLF after major hepatectomy for HCC, this predictive model might serve as a quantitative scoring system to individually estimate the risk of PHLF and to select HCC patients for major hepatectomy.

PHLF was one of the most common complications and the leading cause of mortality after PH, particularly in patients who underwent major hepatectomy (3–7, 24). In the present study, 155 (22.5%) and 37 (19.3%) patients in the training and validation cohorts met the ISGLS criteria for PHLF, respectively. Among them, 93 (13.5%) and 18 (9.4%) patients experienced ISGLS grade B/C PHLF, respectively. As patients with grade A PHLF have a postoperative deterioration that does not require a changing of clinical management and is not associated with increase in perioperative mortality, we defined ISGLS grade B or C as the study endpoint. Our results revealed an overall PHLF-related mortality of 59.5%, which addressed the importance of preoperatively accurate prediction of PHLF for decision-making of treatment and postoperative management among patients undergoing major hepatectomy.

Previously, several models were established to predict PHLF but exhibited obvious heterogeneity. Some nomograms were derived from data of patients with benign and malignant lesions (15), while some nomograms incorporated both preoperative and intraoperative variables (15, 25), and some nomograms were based on analysis of both minor and major hepatectomies (17, 18, 26). Recently, Chin and colleagues (27) created a PHLF prognostic nomogram for major hepatectomy; however, the study involved patients with either HCC or colorectal liver metastasis. Dhir et al. (24) also developed a nomogram only using preoperative factors, but their model was based on data of the West HCC population. Meanwhile, most of the proposed nomograms lack external validation. In this study, the established nomogram was based on data from HCC patients who all experienced major hepatectomy; its predictive ability was validated by an external cohort and showed better performance in the prediction of grade B/C PHLF when compared with the MELD, ALBI, and Child–Pugh scores. The nomogram might be helpful for preoperative prediction of grade B/C PHLF before major hepatectomy.

In the present study, age, gender, TBIL, PT, and CSPH were identified as independent predictors of severe PHLF. The previous study had revealed that advanced age (≥65 years) increased the risk of PHLF through mechanisms involving altered immune response, predisposition to sepsis, and limited regenerative capacity of hepatocytes (28). Similarly, our results also indicated that advanced age had a higher risk of grade B/C PHLF. Interestingly, our results suggested that male gender was an independent risk factor of grade B/C PHLF. This result was similar to the findings noted by John BV and colleagues (29). The exact mechanisms for the increased prevalence of severe PHLF in men are unclear. Male subjects had higher MELD score, higher TBIL and ALT, and longer PT at baseline (**Appendix Table 1**), which may explain the positive association of male and PHLF in the current study.

Total bilirubin and prothrombin time were important indicators of liver function, and CSPH due to liver cirrhosis is characterized by gradual replacement of normal hepatic parenchyma and results in decreased levels of hepatocyte regeneration and growth after major liver resection, which translates to a high mortality rate and increased risk of PHLF. European Association for the Study of the Liver (EASL) (30) and American Association for the Study of Liver Diseases (AASLD) (22) guidelines consider CSPH to be a relative contraindication to liver resection because of the high risk of postoperative liver decompensation. Our results showed that CSPH was an independent risk factor of PHLF which indicated that more assessments should be made upon these high-risk patients before surgery.

The strengths of this study lie in the large patient population and a positive external validation. However, the current study has several limitations. First, it is a retrospective study with its natural defects. Second, the majority of the enrolled HCC patients were related to HBV; whether the nomogram can be used upon patients from the West is still uncertain because the underlying liver diseases in HCC patients are quite different between the East and West. Therefore, the nomogram requires validation in the West patient population. Finally, as the type of resection of all patients was major hepatectomy, we did not assess the impact of FLR volume on PHLF. Therefore, further external validation of the proposed nomogram should be carried out in the future studies.

## Conclusion

In summary, by incorporating five essential preoperative parameters (age, sex, TBIL, PT, CSPH), a nomogram for individualized prediction of ISGLS grade B/C PHLF in HCC patients who underwent major hepatectomy was developed and showed better predictive accuracy than the MELD and ALBI scores. The nomogram could serve as a convenient tool to select proper candidates of major hepatectomy and improve postoperative surveillance.

## Data Availability Statement

The raw data supporting the conclusions of this article will be made available by the authors, without undue reservation.

## Ethics Statement

The studies involving human participants were reviewed and approved by Institutional Ethics Committees of the Eastern Hepatobiliary Surgery Hospital and Zhongda Hospital. The patients/participants provided their written informed consent to participate in this study. Written informed consent was obtained from the individual(s) for the publication of any potentially identifiable images or data included in this article.

## Author Contributions

Conception and design: ZC, ZL. Financial support: ZL, ZC. Administrative support: ZC. Provision of study materials or patients: ZC, ZL, AS, PY, XW. Collection and assembly of data: all authors. Data analysis and interpretation: all authors. Manuscript writing: all authors. All authors contributed to the article and approved the submitted version.

## Funding

This study was supported by the National Natural Science Foundation of China (81871988 to ZC and 82002584 to ZL) and the Jiangsu Province Key Research and Development Program (BE2019747 to ZC).

## Conflict of Interest

The authors declare that the research was conducted in the absence of any commercial or financial relationships that could be construed as a potential conflict of interest.

## Publisher’s Note

All claims expressed in this article are solely those of the authors and do not necessarily represent those of their affiliated organizations, or those of the publisher, the editors and the reviewers. Any product that may be evaluated in this article, or claim that may be made by its manufacturer, is not guaranteed or endorsed by the publisher.

## Supplementary Material

Supplementary Figure 1 Variable clustering plot according to the squares of the Spearman correlation coefficients.

Supplementary Figure 2 Predictor selection based on least absolute shrinkage and selection operator (LASSO) regression analysis of grade B/C PHLF in the training cohort. **(A)** λ selection in the LASSO analysis used 10-fold cross-validation by minimum criteria for mean-square error. Dotted vertical lines were drawn at the optimal values (λ = 0.013) by using the minimum criteria. **(B)** LASSO coefficient profiles of the 18 preoperative variables. A coefficient profile plot was produced against the log λ sequence.

## Abbreviations

HCC, hepatocellular carcinoma; PH, partial hepatectomy; PHLF, post-hepatectomy liver failure; MELD, model for end-stage liver disease; ALBI, albumin–bilirubin; AUROC, areas under the receiver operating characteristic curves; EHBH, Eastern Hepatobiliary Surgery Hospital in Shanghai; ZDH, Zhongda Hospital; QHMD, Qin Huai Medical District of Jinling Hospital; PVE, preoperative portal vein embolization; ALPPS, associating liver partition and portal vein ligation; TACE, transcatheter arterial chemoembolization; PTCD, percutaneous transhepatic cholangial drainage; ENBD, endoscopic nasobiliary drainage; CT, computed tomography; MRI, magnetic resonance imaging; FLR, future liver remnant; AFP, alpha-fetoprotein; HBV-DNA, HBV deoxyribonucleic acid; ISGLS, International Study Group on Liver Surgery grading; CSPH, clinically significant portal hypertension; LASSO, least absolute shrinkage and selection operator; AST, aspartate aminotransferase; ALT, alanine aminotransferase; HBsAg, hepatitis B surface antigen; HBeAg, hepatitis B e antigen; PT, prothrombin time; INR, international normalized ratio; TBIL, total bilirubin; ALB, albumin; PLT, platelet count; anti-HCV, hepatitis C virus antibody; AIC, Akaike information criterion; EASL, European Association for the Study of the Liver (EASL); AASLD, American Association for the Study of Liver Diseases.
