# Supplementary figures and images for: A Novel Nomogram for Predicting Postoperative Liver Failure After Major Hepatectomy for Hepatocellular Carcinoma
Source: Front Oncol. 2022 Mar 14;12:817895. doi: 10.3389/fonc.2022.817895 (PMC8964030; doi:10.3389/fonc.2022.817895)

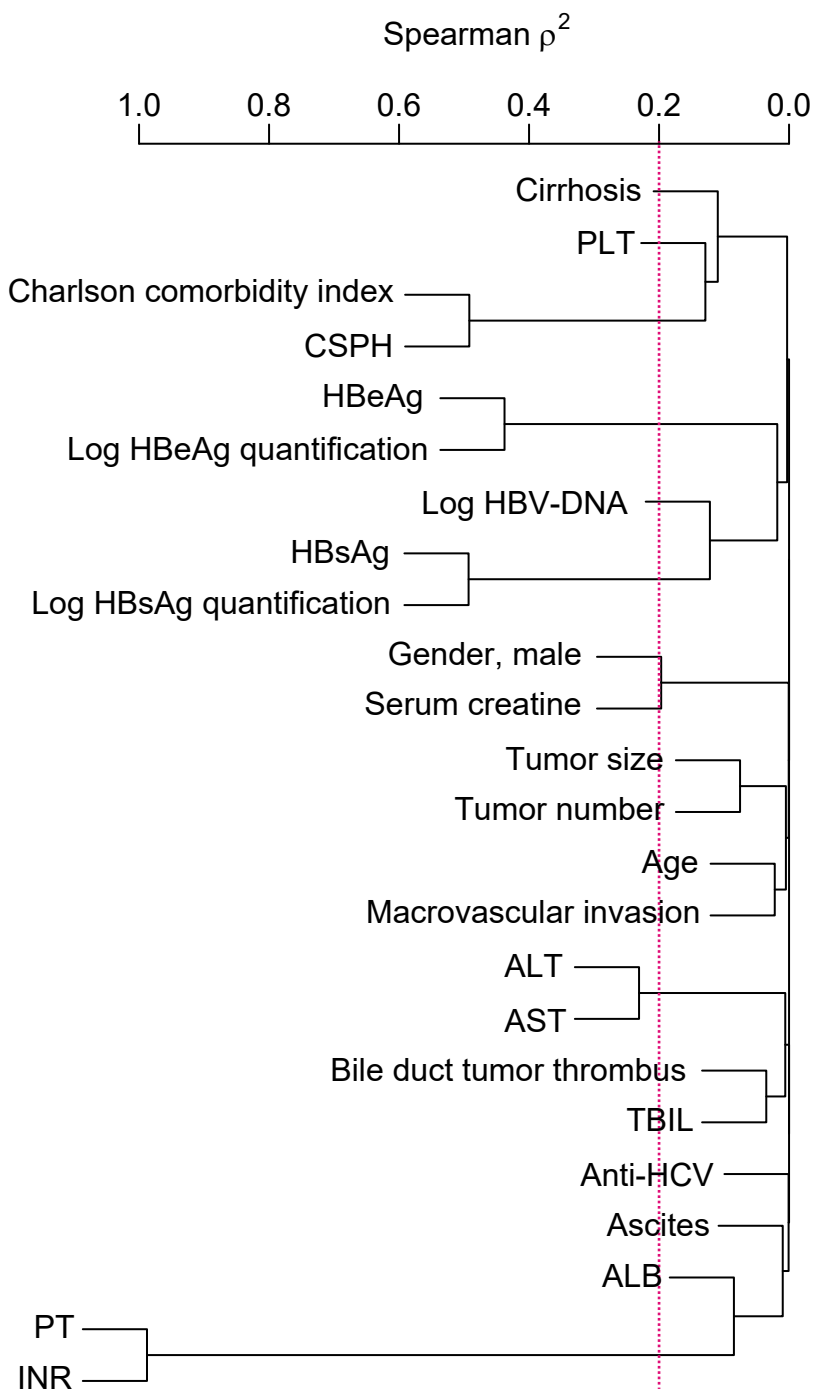

Supplement: Supplementary Figure 1 — Variable clustering plot according to the squares of the Spearman correlation coefficients. [file DataSheet_1.pdf]

**A**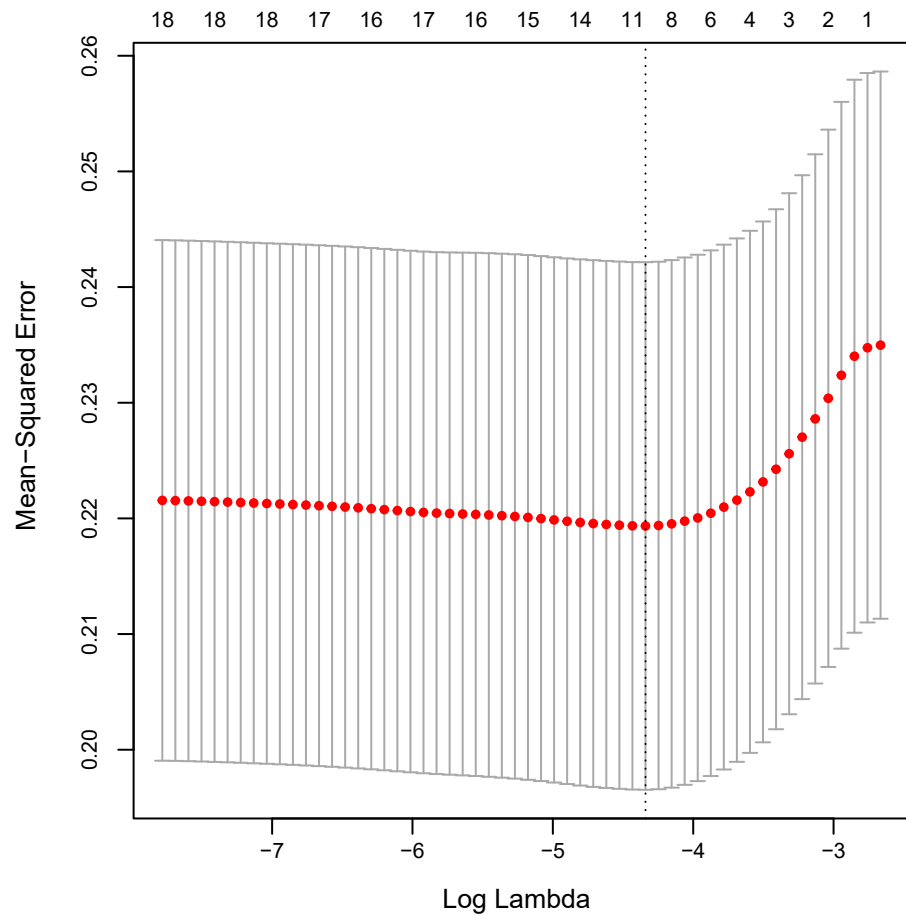**B**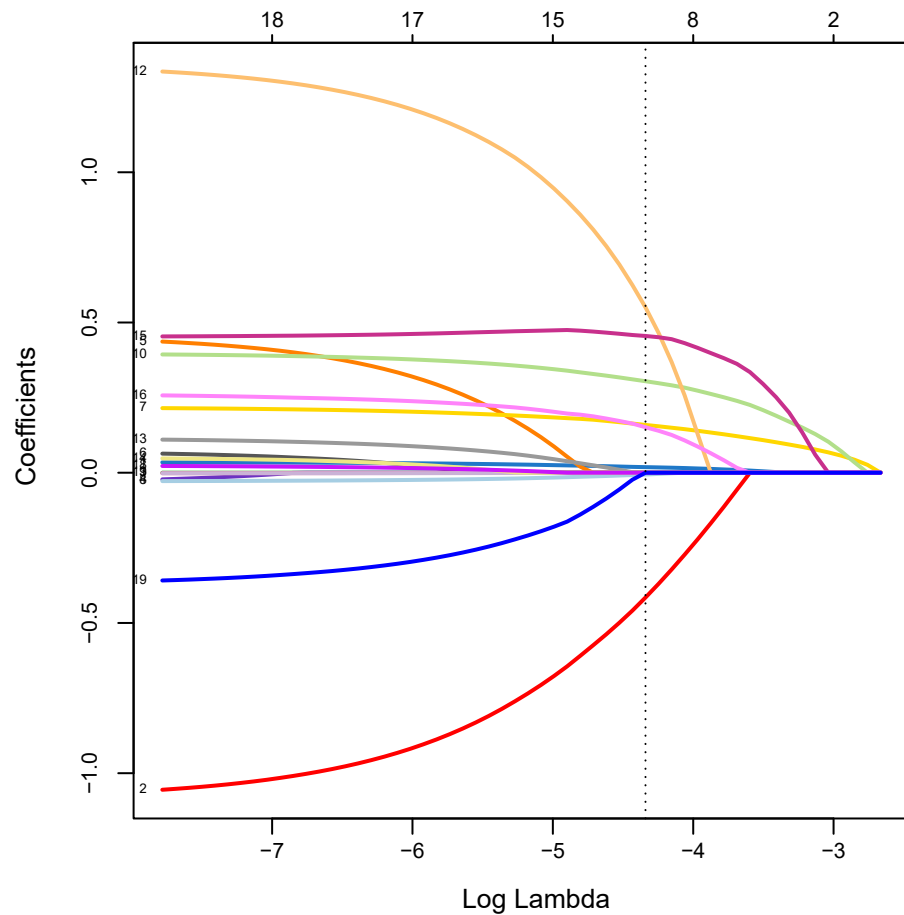

Supplement: Supplementary Figure 2 — Predictor selection based on least absolute shrinkage and selection operator (LASSO) regression analysis of grade B/C PHLF in the training cohort. (A) λ selection in the LASSO analysis used 10-fold cross-validation by minimum criteria for mean-square error. Dotted vertical lines were drawn at the optimal values (λ = 0.013) by using the minimum criteria. (B) LASSO coefficient profiles of the 18 preoperative variables. A coefficient profile plot was produced against the log λ sequence. [file DataSheet_2.pdf]
